# Understanding the intention-behavior gap: The role of intention strength

**DOI:** 10.3389/fpsyg.2022.923464

**Published:** 2022-08-04

**Authors:** Mark Conner, Paul Norman

**Affiliations:** ^1^School of Psychology, University of Leeds, Leeds, United Kingdom; ^2^Department of Psychology, University of Sheffield, Sheffield, United Kingdom

**Keywords:** intention, intention strength, intention-behavior gap, attitude strength, intention stability, physical activity, health behavior

## Abstract

This manuscript overviews recent research on the intention-behavior gap, focusing on moderators of the intention-behavior relationship. The manuscript draws on the concept of intention strength to make two important points. First, strong intentions provide better predictions of behavior, thereby reducing the intention-behavior gap. However, strong intentions have the additional features of being more stable over time, less pliable in the face of interventions to change them, and more likely to bias information processing about engaging in the behavior. These four features of intention strength are not independent. For example, stable intentions are likely to provide better predictions of behavior. Second, various predictors of strength (e.g., importance, certainty, extremity) may also constitute important, but little studied, moderators of the intention-behavior relationship. Moreover, the effects of these moderators of the intention-behavior relationship may be mediated through intention stability (and perhaps other features of intention strength). Future research on the intention-behavior gap would benefit from a more systematic consideration of a broad range of moderators of the intention-behavior relationship both individually and in combination. In addition, future research could usefully explore how these moderating effects might be explained. Such a systematic approach may further our understanding of the intention-behavior gap in relation to physical activity and other behaviors.

## Introduction

Definitions of intentions often focus on the idea of them being self-instructions (Triandis, 1980; Sheeran and Webb, 2016) that capture the underlying motivation (Rogers, 1983) or commitment (Sheeran and Webb, 2016) to act. It is common to distinguish goal (e.g., “I intend to get fit”) and behavioral (e.g., “I intend to engage in physical activity at least five times per week”) intentions, with the former focusing achieving desired goals and the latter focusing on engaging in a behavior or action (perhaps in the service of reaching a goal). It is the latter that are the main focus here. Behavioral intentions are central to a range of theories about the determinants of behavior/action. Indeed they are the proximal and sole determinant of action in the reasoned action approach (Fishbein and Ajzen, 2010) and in protection motivation theory (Rogers, 1983), and one of several proximal determinants of behavior in social cognitive theory (Bandura, 1997) and the health action process approach (Schwarzer and Luszczynska, 2015). Nevertheless, intentions rarely if ever explain *all* the variance in behavior. This has become known as the intention-behavior gap and is the focus of the current manuscript.

**FIGURE 1:**
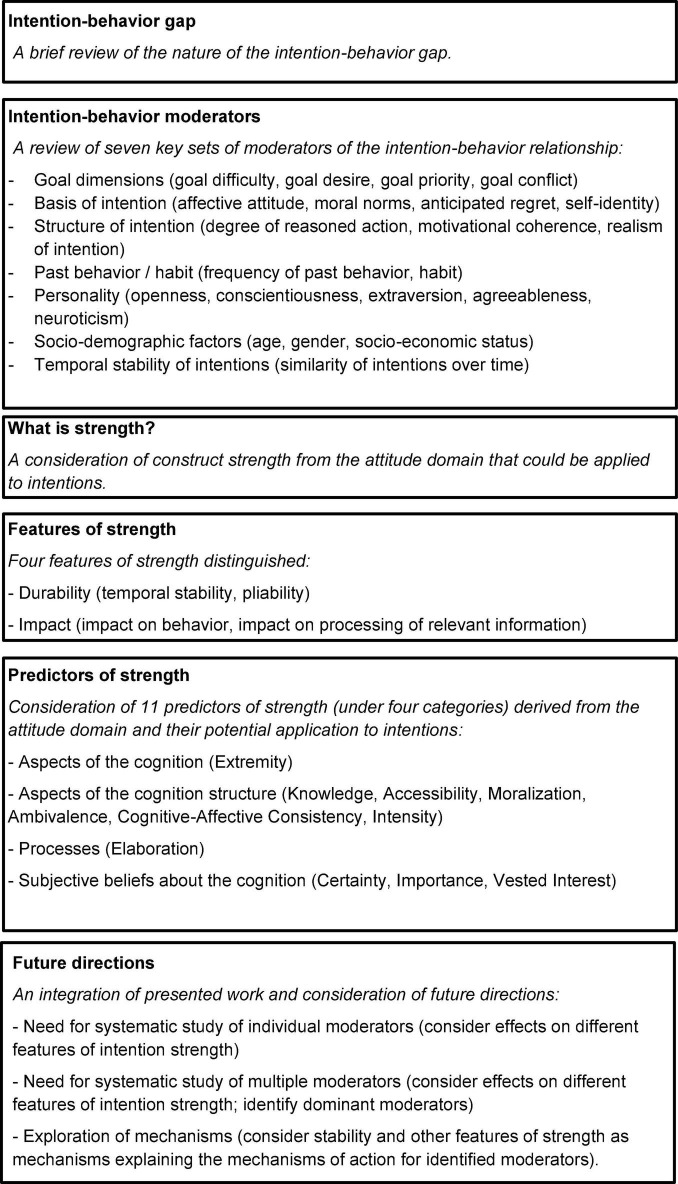
Route map and summary of main sections of the manuscript.

The current manuscript is divided into six main sections (see Figure 1 for a route map and summary). The first briefly reviews the intention-behavior gap for physical activity and other behaviors. The second reviews work on various factors that reduce the intention-behavior relationship (i.e., moderate the intention-behavior relationship) for physical activity and other behaviors. The third introduces the concept of intention strength as a means to improve our understanding of the intention-behavior relationship. The fourth and fifth consider features and then predictors of intention strength. In particular, these sections consider the intention-behavior gap as one of four key features of intention strength and reviews various predictors of intention strength as potential moderators of the intention-behavior relationship. The sixth and final section discusses a number of directions for future research on the intention-behavior gap.

## Intention-behavior gap

Measures of behavioral intentions (e.g., “I intend to engage in physical activity at least five times per week,” strongly disagree – strongly agree) commonly include both a valence (i.e., intenders versus non-intenders and sometimes a neutral category) and an extremity component (i.e., slightly agree versus strongly agree) component. Such measures of intention to engage in a behavior rarely predict all or even the majority of the variance in behavior. For example, reviews of the theory of planned behavior and the reasoned action approach indicate that intentions explain between 18 and 23% of the variance in behavior across a broad range of behaviors (Armitage and Conner, 2001) or health behaviors in particular (McEachan et al., 2011, 2016). Reviews suggest values at the lower end of this range for studies focusing on physical activity (18% variance in Hagger et al., 2002; 20% in McEachan et al., 2011). It is notable that the (mostly prospective) correlational studies included in these reviews almost exclusively focus on behavioral intentions rather than goal intentions. Experimental studies that manipulate intentions and observe effects on subsequent behavior similarly indicate less than perfect relationships. For example, studies of a range of behaviors indicate that medium-to-large sized changes in intentions are associated with only small-to-medium sized changes in behavior (Webb and Sheeran, 2006; Sheeran et al., 2016). Similar sized effects are reported in studies focusing just on physical activity (Rhodes and Dickau, 2012). It is notable that the effect sizes reported in reviews of these experimental studies (*r*_+_ = 0.08–0.18) are considerably smaller than those reported in reviews of correlational studies (*r*_+_ = 0.40–0.48). The disjunction between intentions and behavior observed in both correlational and experimental studies has been termed the intention-behavior gap (Sheeran, 2002; see also Godin and Conner, 2008; Rhodes and de Bruijn, 2013).

A range of methodological factors will be associated with either a narrowing or widening of this gap; more reliable measures of intention and behavior or a focus on relationships disattenuated for measurement error may be associated with a narrowing of the gap, while a focus on objective measures of behavior and failure to ensure that measures of intention and action/behavior are matched on the principle of correspondence (Ajzen, 1991) may widen the gap. Unfortunately the former effects may be more modest than the latter effects. For example, McEachan et al. (2011) estimated the intention-behavior correlation increased from 0.40 to 0.43 when disattentuated for measurement error. In contrast, McEachan et al. (2011) reported that intention and perceived behavioral control explained 26% of the variance in self-reported measures of physical activity, but only 12% of the variance in objective measures. Across a broader set of behaviors, Armitage and Conner (2001) reported that intention and perceived behavioral control explained 31% of the variance in self-reported behaviors, but only 20% of the variance in objective behaviors. Matching of intention (plus other measures) and behavior measures on action and target elements is central to the theory of planned behavior (Ajzen, 1991). However, some behaviors such as physical activity often employ frequency measures of behavior that commonly fail to follow this principle. For example, some physical activity studies measure behavior based on frequency measures such as the International Physical Activity Questionnaire (sometimes converting this into METs) but predict this from intention items focusing on meeting recommended guidelines for physical activity (e.g., five sessions per week of at least 20 mins). The problem here is that the intention measure should be matched to a behavior item that taps whether the recommended activity had been completed or not rather than number of METs. The linearity of the relationship between such an intention measure and a measure of METs is unclear and could under- or over-estimate the size of the true intention-behavior relationship (see Morwitz and Munz, 2020 on other methodological issues in relation to the intention-behavior relationship).

An important focus in furthering our understanding of the intention-behavior gap has been exploring the role of conceptual moderators. This research focuses mainly on features of the intention and other constructs that influence the intention-behavior gap and is briefly reviewed in the next section.

## Intention-behavior moderators

In the existing research literature, a key approach to understanding the intention-behavior gap in relation to physical activity and other behaviors has been to focus on potential moderators of the relationship, i.e., factors associated with changes in the magnitude of the intention-behavior relationship. Moderators help identify the limits of the relationship between intention and behavior and also the conditions under which strong versus weak relationships might be expected. A range of such moderators have been identified in various studies and discussed in several reviews (e.g., Sheeran, 2002; Sheeran and Webb, 2016). In addition, a recent, broad review of intention-physical activity moderators (Rhodes et al., 2022) provides a number of insights. The moderators that have received the most attention can be broadly split into goal dimensions, basis of intention, structure of intention, past behavior/habit, personality and socio-demographic factors, and temporal stability.

### Goal dimensions

The goal dimensions explored as moderators of the intention-behavior relationship include goal difficulty, goal desire and commitment, plus goal priority and conflict. As the difficulty of a goal increases, the power of intentions to predict behavior decreases (e.g., Sheeran et al., 2003). Goal difficulty is a function of the goal itself and the skills, resources, and effort an individual can bring to achieving the goal. Measures typically tackle how easy or difficult the individual perceives it to be to achieve the goal. Easy-difficult judgments are a key component of perceived behavioral control. Lower perceived behavioral control (i.e., high goal difficulty) should be associated with larger intention-behavior gaps. This is the prediction in the theory of planned behavior and reasoned action approach (Fishbein and Ajzen, 2010). However, the empirical findings are mixed. Armitage and Conner (2001) reported that approximately 50% (9/19 studies) of the studies in their meta-analysis testing the interaction between perceived behavioral control and intentions reported a significant effect (i.e., intentions being stronger predictors when perceived behavioral control was higher). More recently, Hagger et al. (2022) reported that across 36 tests, the intention-behavior relationship was stronger when perceived behavioral control was high (M + 1SD: b = 0.555, 95%CI [0.452, 0.658]) compared to moderate (M: b = 0.489, 95%CI [0.384, 0.594]) or low (M-1SD: b = 0.423, 95%CI [0.301, 0.545]). Rhodes et al. (2022) in their review reported that perceived behavioral control significantly moderated the intention-behavior relationship for physical activity in approximately 60% (13/21 tests) of studies reporting this effect. Sheeran and Webb (2016) argue that some of the mixed findings for perceptions of goal difficulty as a moderator may be attributable to people under-estimating actual difficulty of performing these more difficult behaviors.

Goal desire and commitment have received much less attention. The more a goal is desired (Prestwich et al., 2008) and the more an individual is committed to the goal (Rhodes et al., 2018) then the stronger should be the intention-behavior relationship. Prestwich et al. (2008) reported stronger intention-behavior relationships at higher compared to lower levels of goal desire across four studies. Greater commitment to a goal might also be expected to increase the intention-behavior relationship (Cooke and Sheeran, 2013). Rhodes et al. (2022) reported that commitment significantly moderated the intention-behavior relationship for physical activity in approximately 70% (2/3 tests) of studies.

Goal conflict and goal priority have also received attention as moderators of the intention-behavior relationship. The less a focal goal conflicts with other goals might be expected to be associated with greater effort to achieve the focal goal and so stronger intention-behavior relationships. Rhodes et al. (2022) reported that goal conflicts significantly moderated the intention-behavior relationship for physical activity in approximately 70% (6/9 tests) of studies reviewed. Goal priority is an important concept in understanding the pursuit of multiple goals and refers to the temporary increase in the importance attached to, and resources directed toward, one or more goals compared to other goals – that serve to benefit the performance of the prioritized behavior (Unsworth et al., 2014). Although conceptually important, goal priority has not received widespread attention as an intention-behavior moderator (Geers et al., 2009; Conner et al., 2016a). Conner et al. (2016a) showed that goal priority moderated the intention-behavior relationship for physical activity (Study 1) and a range of health behaviors (Study 4) and that a manipulation of goal priority increased the intention-physical activity relationship when physical activity was self-reported (Study 2) or objectively measured (Study 3). More recently, Conner et al. (2022c) showed that prioritizing one or two behaviors (including physical activity) that individuals intended to engage in resulted in greater performance of the prioritized behaviors with no decrement to the non-prioritized behaviors.

### Basis of intention

Studies that have explored the basis of intention as a moderator of the intention-behavior relationship have primarily considered the extent to which intentions are based on affect or identity. Several studies indicate that intentions based more on personal or affective compared to other factors are more predictive of behavior, consistent with the predictions of self-determination theory (Deci and Ryan, 1985). This includes work on attitudes versus norms (Sheeran et al., 1999) and affective versus instrumental attitudes (Keer et al., 2014). Similarly, high levels of moral norms (Godin et al., 2005), anticipated regret (Sheeran and Abraham, 2003), and self-identity (Sheeran and Orbell, 2000; Carfora et al., 2017) have also been found to be associated with stronger intention-behavior relationships. Rhodes et al. (2022) reported that affective attitudes (4/6 tests), anticipated regret (4/5 tests), and physical activity personal/self-identity (5/7 tests) each significantly moderated the intention-physical activity relationship in their review. Across a group of health behaviors including physical activity, Conner et al. (2016b) showed that out of instrumental attitude, affective attitude, injunctive norm, descriptive norm, and anticipated regret, it was intentions based on anticipated regret that most strongly predicted behavior. Other studies have shown that drawing attention to anticipated regret via measuring it (drawing on the Question-Behavior Effect; Wood et al., 2016) is sufficient to increase the power of intentions to predict behavior. For example, Sandberg and Conner (2011) showed that measuring anticipated regret increased the power of intentions to predict objectively measured sports center use. Sandberg and Conner (2009) showed similar effects for objective measured attendance for cervical screening. Importantly this effect was only present when anticipated regret was assessed before rather than after intention, suggesting that the anticipation of regret had to inform the intention for the moderation effect to occur.

### Structure of intention

A number of recent studies have explored various aspects of the structure of intention as moderators of the intention-behavior relationship. This includes work on the degree of reasoned action (Sheeran and Conner, 2019), motivational coherence (Sheeran and Conner, 2017) and the realism of the intention (Avisha et al., 2019). Degree of reasoned action refers to the extent to which a person’s determination to act is based on relevant expectancies, or how well behavior-relevant cognitions predict intentions. Across two studies, Sheeran and Conner (2019) showed that well-reasoned intentions better predicted behavior. Motivational coherence is the extent to which predictors of intentions (e.g., attitudes, norms, perceived control from the theory of planned behavior) cohere or point in the same direction. Across three studies (including one on physical activity), Sheeran and Conner (2017) showed that greater motivational coherence was associated with a stronger relationship between intentions and behavior. Finally, Avisha et al. (2019) examined how realistic intentions (i.e., those based on considerations of the expectations that the behavior could be performed) might moderate the intention-behavior relationship. Across three studies (including one on physical activity), it was shown that more realistic intentions were stronger predictors of behavior.

### Past behavior/habit

The frequency of past performance of a behavior (or habit based on self-report measures) has also been found to moderate the intention-behavior relationship, although the direction of this moderation effect is inconsistent. For example, Rhodes et al. (2022) reported that past behavior/habit was associated with a significantly stronger intention-physical activity relationship in approximately 30% (4/14 tests) of studies, but a significantly weaker relationship in approximately 50% (7/14 tests) of studies (see also Gardner et al., 2011). Sheeran et al. (2017) showed that this apparent inconsistency can be explained by the fact that the impact of past behavior/habit on the relationship between intentions and behavior follows an inverted U-shaped relationship. At low levels of past behavior, increasing experience initially enhances the power of intention to predict behavior, while at higher levels of past behavior increasing experience attenuates the power of intention to predict behavior. The former effect may be attributable to experience strengthening the intention (similar arguments have been made in relation to experience on the attitude-behavior relationship; Fazio and Zanna, 1978), while the latter effect may be attributable to the behavior becoming more automatized or habitual (Ouellette and Wood, 1998).

### Personality

The personality factors explored as moderators of the intention-behavior relationship have included all of the big five personality dimensions (openness, conscientiousness, extraversion, agreeableness, and neuroticism) with generally no significant effects (see Rhodes et al., 2022). The exception to this trend is conscientiousness, with Rhodes et al. (2022) reporting that 80% (4/5 tests) of studies reported that higher levels of conscientiousness were associated with stronger intention-physical activity relationships. Conscientiousness refers to the ability to control one’s behavior and to complete tasks, with individuals high in conscientiousness being more organized, careful, dependable, self-disciplined and achievement-oriented than those low in conscientiousness (McCrae and Costa, 1987). In addition, conscientiousness is associated with greater impulse control (Bogg and Roberts, 2004, 2013). A number of these factors might help explain why those high in conscientiousness show stronger relationships between their intentions and behavior with perhaps different factors operating in relation to risk (e.g., impulse control) versus protection (e.g., self-discipline) behaviors like physical activity. These explanations remain to be tested.

### Socio-demographic factors

A number of socio-demographic factors have been explored as moderators of the intention-behavior relationship with mostly null effects. For example, Rhodes et al. (2022) reviewed a range of such demographic factors including age and gender although no clear evidence emerged of consistent effects across the various physical activity studies examined. One exception to these generally null effects has been socio-economic status. A number of studies on physical activity (Schüz et al., 2017) and other behaviors (e.g., Conner et al., 2013; Schüz et al., 2020, 2021) indicate that socio-economic status moderates the intention-behavior relationship (i.e., weaker intention-behavior relationships in lower SES groups). However, Rhodes et al. (2022) reported mixed effects for different measures of socio-economic status on intention-physical activity relationships: 1/2 significant effects for material deprivation, 1/5 significant effects for income, 0/1 for social deprivation, and 2/4 for education. The observed effects may be attributable to the same goal being of greater difficulty in lower socio-economic status groups due to variations in the opportunities, resources, ability, skills and time and effort required to realize the goal (Sheeran and Webb, 2016; Schüz, 2017).

### Temporal stability of intentions

Research in the goal/behavioral intention domain has also focused on the whether the temporal stability of intentions is associated with stronger effects on behavior. Temporal stability here refers to the lack of change in an intention measure over time. In most studies this is typically operationalized as a lack of absolute change in an intention measure within an individual over time (see Conner et al., 2000 for consideration of different measures of stability). For intensive longitudinal designs, where intention is measured multiple times, stability might be better captured by some form of within-person variability measure (with low variability equating to greater stability). The important moderating role of temporal stability has been highlighted as one of the limiting conditions of the Theory of Planned Behavior/Reasoned Action and Reasoned Action Approach (Fishbein and Ajzen, 2010) which states that intentions will only predict behavior to the extent that they remain unchanged between when they are measured and the time point at which they may influence the decision to act. A number of studies show that more stable intentions better predict behavior. Cooke and Sheeran’s (2004) meta-analysis showed temporal stability to significantly moderate intention-behavior (10 studies) relationships. More specifically in relation to physical activity, Conner and Godin (2007), across seven studies, showed the intention-behavior relationship to be a substantial *r*_+_ = 0.60 when intentions were stable, but only *r*_+_ = 0.27 when intentions were unstable. Similarly, the review of Rhodes et al. (2022) showed intention stability to be a significant moderator of intention-physical activity relationships in nearly 80% (10/13 tests) of studies and noted intention stability as one of the more consistent moderators. Beyond the physical activity domain, Conner et al. (2002) reported intentions were stronger predictors of healthy eating over a period of 6 years when these intentions were stable over a 6-month time period. More recently, Norman et al. (2022) showed that the temporal stability of intentions moderated intention-behavior relationships across a number of COVID-19 protection behaviors. As discussed in subsequent sections, the temporal stability of intentions may also be considered a key feature of a strong intention and also represent a key mechanism to explain the effects of other moderators of the intention-behavior relationship.

## What is strength?

The moderators reviewed in the previous section provide a number of insights into the factors that may account for the intention-behavior gap. However, in general they fail to provide a strong framework for understanding the magnitude of the impact of intentions on behavior. The subsequent sections of this manuscript consider the concept of *intention strength*, what it can add to our understanding of the intention-behavior relationship, and how it might provide the basis for such a framework.

The concept of “strength” in relation to social/health cognitions has received the most attention in relation to attitudes. Attitude strength has been defined as “the extent to which attitudes manifest the qualities of durability and impactfulness” (Petty and Krosnick, 1995, p. 3). Thus, strong attitudes are stable and resistant to efforts to change them (i.e., they are durable) and they bias information processing and guide behavior (i.e., they are impactful). In the attitude literature, a distinction is made between predictors versus the defining features of attitude strength (Luttrell and Sawicki, 2020). Predictors of attitude strength include the importance, accessibility and extremity of an attitude, while defining features include the attitude’s temporal stability and impact on behavior.

The idea that goal or behavioral intentions also possess a dimension of strength has received comparatively less attention, although the idea does appear sporadically in the literature. For example, Hall (2013), in the *Encyclopedia of Behavioral Medicine*, offers this definition:

“Intention strength can be defined as the quantity of personal resources that an individual is prepared to invest in executing a behavior. Intention strength is closely akin to the concept of “motivation,” with high levels of intention strength understood to represent strong motivation to perform a behavior.”

Similarly, Fuchs et al. (2017) suggest that “intention strength refers to the degree of firmness a person expresses toward an intended action” and Rebar et al. (2019) define intention strength as “the degree of commitment a person has to enact their intention.” The distinction here is between the focus of the intention and the strength of the commitment to pursue that intention. Rebar et al. (2019) label these decisional intentions versus intention strength. Decisional intentions can be tapped by items such as “I intend to engage in __ minutes of physical activity next week,” yes/no. In contrast, strength of commitment can be tapped by items such as “How strong is your intention to resume your fitness training within the next weeks and months?,” I do not have this intention at all – I do have a very strong intention (Fuchs et al., 2017), or “To what degree do you intend to engage in physical activity next week?,” Very little – Very much (Rebar et al., 2019).

These definitions of strength identify some of the different predictors of strength but say little about the consequences of having a strong intention. It is argued here that work on intention strength would benefit from employing a similar definition to that used for attitude strength. That is, intention strength should be broadly defined in terms of the extent to which intentions manifest the qualities of durability and impactfulness. These are discussed in the next section under the features of intention strength. A subsequent section examines a number of predictors of strength that might be expected to impact on the features of strength.

## Features of strength

There are interesting parallels between work on the intention-behavior gap and work on the attitude-behavior relationship. In the attitude domain, strong attitudes are defined as having the consequences of being durable and having impact (Petty and Krosnick, 1995). Luttrell and Sawicki (2020) refer to these as the *defining features* of attitude strength. Durability can be further split into temporal stability and pliability (or persistence and resistance), while impact can be further split into effects of the attitude on behavior and the processing of attitude-relevant information. Temporal stability and impact on behavior (i.e., the attitude-behavior gap) are the defining features of attitude strength that have received the most attention (Petty and Krosnick, 1995). It is worth noting that these two features of strong attitudes are not unrelated, with attitude temporal stability being one important mechanism through which strong attitudes better predict behavior (the prediction explanation; Fabrigar et al., 2005). As Schwartz (1978) noted, attitudes will not be likely to predict subsequent behavior unless they persist over the intervening time interval between when the two are measured. A number of previous studies support this prediction explanation (Schwartz, 1978; Davidson and Jaccard, 1979; see also Glasman and Albarracín, 2006). More recently, Conner et al. (2022a) showed across three studies that more stable attitudes were more predictive of subsequent behavior. Indeed, temporally stable attitudes may predict behavior over periods as long as 10 years (Conner and Norman, 2021). Research has also looked at the resistance of strong attitudes to persuasive attempts and the impacts of strong attitudes on information processing. In general, this research shows that stronger attitudes are more resistant to efforts to change them (Eagly and Chaiken, 1995) and have greater impact on the processing of attitude-relevant information (Petty and Krosnick, 1995) leading to more biased processing (i.e., enhancement of information consistent with current attitude and denigration of information inconsistent with current attitude).

As with strong attitudes, strong intentions might usefully be defined as having the consequences of being durable and having impact. Durability can be split into the temporal stability of intentions and the pliability of intentions. Impact can be split into effects of the intention on behavior and on the processing of intention-relevant information. In the intentions domain it is the intention-behavior relationship that has received the most attention. From an intention strength perspective, strong intentions are more predictive of behavior and therefore reduce the gap between intentions and behavior. However, strong intentions are also likely to have the features of being stable over time, less pliable when challenged, and having greater impacts on the processing of intention relevant information. The need for these features of intention strength to be given more attention alongside examination of impacts on the intention-behavior relationship is noted in the future directions section below. Importantly stability, pliability and information processing effects may each represent important mechanisms by which moderators of the intention-behavior relationship have their effects.

## Predictors of strength

A number of factors may be associated with having strong cognitions. In the attitude domain, Luttrell and Sawicki (2020) refer to these as *predictors* of attitude strength. In relation to predictors of attitude strength, Howe and Krosnick (2017) identified 11 predictors: certainty, importance, ambivalence, accessibility, knowledge volume, extremity, cognitive-affective consistency, intensity, moral conviction, elaboration, and vested interest. Similarly, Luttrell and Sawicki (2020) identified seven such predictors: accessibility, ambivalence, certainty, importance, elaboration, knowledge, and moralization.

Many of these predictors of attitude strength may also have direct or indirect utility in relation to understanding intention strength. Each of the eleven predictors of strength identified in previous reviews (Howe and Krosnick, 2017; Luttrell and Sawicki, 2020) are discussed in detail below: extremity, knowledge, accessibility, moralization, ambivalence, cognitive-affective consistency, intensity, elaboration, certainty, importance, and vested interest. Evidence from the attitude strength literature is reviewed alongside any work in the intention domain.

These different predictors of strength fall into one of four basic categories (Petty and Krosnick, 1995; Eaton and Visser, 2008). The first category comprises *aspects of the cognition*. Extremity is the key predictor in this category. The second category comprises *aspects of the cognition structure*. This includes aspects of the structure of the thoughts associated with the cognition in memory such as the amount of knowledge linked to the cognition in memory (i.e., knowledge) and the strength of the association between the cognition and the object (i.e., accessibility), but also the extent to which the cognition is based on something being right or wrong or moral or immoral (i.e., moralization), the extent to which positive and negative evaluations are incongruent (i.e., ambivalence), the extent to which cognitive and affective evaluations are incongruent (i.e., cognitive-affective inconsistency), and the extent to which strong emotions are elicited (i.e., intensity). The third category comprises *processes* by which the cognition is formed. This includes the degree of thinking done (i.e., elaboration) about the merits and shortcomings of target. The fourth and final category comprises the *subjective beliefs about the cognition*. This includes the degree of certainty about the object, the importance given to the cognition or the object, and vested interest in the cognition.

### Aspects of the construct

In relation to *extremity*, attitude measures are typically operationalized using bipolar scales with a neutral mid-point (e.g., “For me, engaging in the recommended levels of physical activity each week over the next month is…bad – good;” scored 1–7). Such measures simultaneously tap the valence of the attitude (i.e., negative for scores 1–3; neutral for a score of 4; positive for scores 5–7) and the extremity of the attitude (i.e., scored as the distance from the neutral point; scores of 5 and 7 both indicate a positive attitude but the latter score indicates a more extreme positive score than the former). More extreme attitudes are assumed to be stronger and considerable literature shows more extreme attitudes to be more predictive of behavior, stable over time, resistant to change and impactful on information processing (see Abelson, 1995 for a review). The majority of tests of the strength of the attitude-behavior relationship employ bipolar measures of attitude that confound the valence and extremity of the attitude. For example, McEachan et al. (2011) meta-analysis of the theory of planned behavior reports an attitude-behavior relationship of *r*_+_ = 0.30 for physical activity based mainly on such bipolar attitude measures. Such analyses assume the attitude-behavior relationship is linear, although an attitude strength perspective might suggest a cubic relationship with the greatest change in behavior apparent at the extremes. Some recent research has supported a cubic relationship between attitude extremity and behavior (Bechler et al., 2021), although here the greatest change in behavior was apparent around the neutral point (these tests mainly focused on the attitude-intention relationship). Bassili (1996) makes the useful distinction between operative and meta-judgmental measures of strength. Operative measures link to processes and may be less open to bias in self-report (e.g., accessibility based on reaction times), while meta-judgmental measures are based on self-perceptions and may be more open to bias in self-report (e.g., perceived importance of a cognition). Extremity measures can be considered to be both operative and meta-judgmental measures of strength.

Similarly in relation to intentions, although few studies explicitly examine extremity, typically measures are bipolar and include elements tapping both direction (equivalent to valence in attitude measures) and extremity which is assumed to tap strength. For example, behavioral intentions toward physical activity might be measured by an item such as, “I intend to engage in the recommended levels of physical activity each week over the next month, strongly disagree – strongly agree” (scored 1–7). Such a measure taps the direction of the intention (i.e., negative/disinclined for scores 1–3; neutral for a score of 4; positive/inclined for scores 5–7) and the extremity of the intention (i.e., scored as the distance from the neutral point; scores of 5 and 7 both indicate a positive intention but the latter score indicates a more extreme [and stronger] positive intention than the former). Although unipolar measures of intention (e.g., “I intend to engage in the recommended levels of physical activity each week over the next month, not at all – definitely;” scored 1–7) are possible, they are relatively little used. For example, the intention-behavior relationship of *r*_+_ = 0.45 for physical activity reported by McEachan et al. (2011) was largely based on bipolar intention measures that include both direction (or valence) and extremity elements. Such analyses assume the intention-behavior relationship is linear, although research points to this being unlikely to be the case (e.g., Rebar et al., 2019). For example, Sheeran (2002) makes the important point that the intention-behavior gap is mainly attributable to those who are inclined to act (i.e., positive intention in the distinction above) failing to subsequently act (i.e., inclined abstainers in his matrix; see also Orbell and Sheeran, 1998). Sheeran (2002) noted that in relation to physical activity, 54% of intenders (i.e., those inclined or with positive intentions) failed to act (and 46% did act), while only 3% of non-intenders (i.e., those disinclined to act/with negative intentions) acted (and 97% failed to act). Rhodes and de Bruijn (2013), in their meta-analysis, reported the overall intention-physical activity gap to be 46% (only 2% of non-intenders acted; while 42% of intenders acted).

Reanalysis of data from Conner et al. (2021; Study 1) on engagement with physical activity examined the effects for both valence/direction and extremity for intention. This study was conducted in a sample of almost 1,000 adults over a 1-month time period using the above bipolar intention measure and a self-report measure of engaging with the recommended level of physical activity (“Over the past month, how many weeks did you engage in the recommended levels of physical activity”? 0–4 weeks; coded into 0–3 weeks non-compliance, 4 weeks compliance). The results indicate that the discordant percentages were 65% for intenders, but only 4% for non-intenders. Table 1 reports the percentage engaging with the behavior for each point on the intention scale. This indicates several interesting findings. First, that the relationship between intention and behavior is not linear (with the pattern either side of the neutral point looking very different). Second, that the relationship between extremity and likelihood of behavior is not linear (even for the positive intender end of the scale). Third, the rate of change in the likelihood of behavior is greater between more extreme positive responses. More detailed examination of how extremity impacts on intention-behavior relationships is warranted, particularly as tests of other moderators of the intention-behavior relationship may be mainly based on measures of intention that include both direction/valence and extremity components. As Bechler et al. (2021) note in relation to the attitude-behavior relationship, different patterns of relationships between extremity and behavior have different implications (e.g., a strength perspective would be expected to lead to an accelerating effect at more extreme levels).

**TABLE 1:** Percentages of respondents reporting engaging in recommended levels of physical activity at different levels of intention (reanalysis of data from Conner et al., 2021, Study 1).

	Scale point						
	1	2	3	4	5	6	7
Behavior	0%	0%	9%	17%	28%	33%	51%

Higher scores indicate more positive intentions (4 is the neutral point).

### Aspects of the construct structure

Attitude *knowledge* or knowledge volume refers to the amount of information the person has about the attitude object. This is usually tapped by knowledge listing tasks or quizzes (i.e., operative indexes), although meta-judgmental measures have also been used. For example, Davidson et al. (1985) asked respondents about how well-informed they were about the attitude object (completely uninformed - completely informed). Davidson et al. (1985) showed greater knowledge to be associated with stronger attitude-behavior relationships and studies have also shown it to be linked to greater attitude stability (Bartle, 2000). Similarly, Conner et al. (2022b) reported that more self-reported knowledge about the behavior was associated with attitudes that were more predictive of behavior.

Knowledge measures typically focus on the attitude object (i.e., behavior) and therefore could also predict the strength of an intention, including its impact on behavior. However, to date there are no studies using operative or meta-judgmental measures of knowledge on the intention-behavior relationship. It might be expected that knowing more about a behavior would be associated with intentions that are more predictive of engaging in the behavior.

In relation to attitudes, *accessibility* is the likelihood that the attitude will come to mind automatically in relevant situations. It is an operative measure (i.e., response latency) and assessed in relation to the evaluation of the attitude object (Fazio, 1995). More accessible attitudes have been found to be more stable over time (Bassili, 1996) and more predictive of behavior (Fazio et al., 1982); they are also more resistant to persuasion (Pfau et al., 2003) and more likely to bias information processing (Houston and Fazio, 1989).

In relation to intentions, accessibility would be the likelihood that the intention comes to mind automatically in relevant situations. It is an operative measure (i.e., response latency) and assessed in relation to the intention. Bassili (1993, 1995) reported more accessible voting intentions for a candidate to better predict voting for that candidate. In contrast, Doll and Ajzen (1992) failed to observe a significant moderating effect for accessibility on intention-behavior relationships in relation to playing with a video game. A meta-analysis by Cooke and Sheeran (2004) reported that across five studies for the intention-behavior relationship that accessibility was a significant moderator. Those with highly accessible intentions (*r*_+_ = 0.75) compared to those with less accessible intentions (*r*_+_ = 0.62) showed stronger intention-behavior relationships. There is a lack of studies testing intention accessibility in relation to engaging in physical activity.

In relation to attitudes, *moralization* or moral conviction is the degree to which an attitude is a strong and absolute belief that something is right versus wrong, moral versus immoral, or that it reflects core moral values and convictions (Skitka, 2014). It is measured by meta-judgmental measures and usually, but not always, measured in relation to the attitude/evaluation (e.g., Skitka, 2014) rather than the object (e.g., Conner et al., 2022b). Various studies have shown such attitudes to be more stable (Luttrell and Togans, 2021) and to better predict behavior (Skitka and Bauman, 2008; Judge et al., 2012). For example, Conner et al. (2022b) showed a measure of moral conviction taken in relation to the object (e.g., “Morally, wearing a face covering in public places is the right thing to do? Strongly disagree-Strongly agree”) in a multi-behavior study significantly moderated the attitude-behavior relationship (i.e., higher moral conviction associated were better predictors of behavior).

To date there is a lack of studies assessing the impact of moralization or moral conviction in relation to the intention or in relation to the behavior on the intention-behavior relationship. As noted earlier, some studies do show that intentions based on moral norms were more predictive of behavior, consistent with this hypothesis. For example, Godin et al. (2005) showed, across six datasets for various behaviors (including physical activity), that intentions more closely aligned with moral norms (compared to attitudes) were more predictive of subsequent behavior. Further tests of moral conviction as a predictor of intention strength, are warranted, although moralization might not be expected to be a key predictor of intention strength in relation to physical activity as physical activity is not typically considered to be a moral behavior.

In the attitude domain, *ambivalence* is the degree to which an individual has both positive and negative reactions to an attitude object (Conner and Sparks, 2002). Greater ambivalence is generally associated with less stable attitudes and weaker attitude-behavior relationships. Cooke and Sheeran (2004) reported a significant effect of ambivalence on the attitude-behavior relationship across six studies, although the average effect size was small. Both meta-judgmental and operative measures of ambivalence are widely used (Conner and Sparks, 2002), although the correlation between the two is modest. A limited number of studies have examined ambivalence as a moderator of the intention-behavior relationship (see Armitage and Conner, 2004), although the effects do not appear to be consistent.

*Cognitive-affective inconsistency* is the absolute difference between the cognitive and affective evaluations of an attitude object (irrespective of whether these evaluations are oppositely valenced or not as would be required for a measure of *cognitive-affective ambivalence*). Conner et al. (2021) found that a measure of cognitive-affective inconsistency, derived from bipolar measures of cognitive and affective attitudes, moderated the attitude-behavior relationship, as did a measure of cognitive-affective ambivalence. Higher levels of cognitive-affective inconsistency and ambivalence were both associated with weaker attitude-behavior relationships, although cognitive-affective inconsistency was the stronger moderator of attitude-behavior relations (Conner et al., 2021). There are few tests of cognitive-affective inconsistency as a predictor of attitude stability (see Chaiken et al., 1995). In addition, to date, there have been no tests of cognitive-affective inconsistency (taken in relation to the behavior) as a moderator of the intention-behavior relationship. It might be expected that when cognitive-affective inconsistency is low intentions to perform the behavior will be more predictive of behavior.

In the attitude domain, *intensity* is the degree to which a person’s evaluation of the attitude object activates powerful emotions (Howe and Krosnick, 2017). Intensity is measured by simple, meta-judgmental measures about how strong the participant’s feelings are about an issue or attitude object (Krosnick and Schuman, 1988). Again, there are no tests to date of intensity taken in relation to the behavior as a moderator of the intention-behavior relationship. It might be expected that when the behavior activates powerful positive emotions then intentions toward to perform the behavior will be more predictive of behavior. For example, if the thought of physical activity elicits powerful positive or negative emotions then intentions might be expected to be more predictive of engaging in physical activity than if no emotions are elicited. This may be related to intentions being better predictors of behavior when based on affective attitudes or anticipated regret. Future research could usefully explore intensity as an intention-behavior moderator in the physical activity domain.

### Cognitive processes

Attitude *elaboration* is the degree of thought or careful consideration one has given to the attitude object’s merits and shortcomings (Barden and Tormala, 2014). The classic measure is based on thought listing where participants list all their thoughts about an attitude object (i.e., operative measures; Petty and Cacioppo, 1977), although meta-judgmental measures of elaboration could be tapped by simple self-report. Studies have shown more elaborated attitudes based on thought-listing to be more stable (Haugtvedt and Petty, 1992) and to better predict behavior (Barden and Petty, 2008). In contrast, Conner et al. (2022b) did not find a meta-judgmental measure of attitude elaboration about the attitude object to moderate the attitude-behavior relationship.

There are no published tests of elaboration (operative or meta-judgmental) taken in relation to the behavior as a moderator of the intention-behavior relationship. It might be expected that greater elaboration about a behavior might lead to intentions that are more predictive of engaging in the behavior.

### Subjective beliefs about the construct

Attitude *certainty* refers to the degree of confidence an individual has that his or her evaluation of the attitude object is correct/clear to him or her. The conviction with which an attitude is held is included as part of other definitions of certainty (Tormala and Rucker, 2018). Simple single-item, meta-judgmental measures are often used to tap certainty (e.g., Fazio and Zanna, 1978) and studies have shown greater certainty to be linked to both greater stability of attitudes (Bassili, 1996) and stronger attitude-behavior relationships (Warland and Sample, 1973; Fazio and Zanna, 1978). Cooke and Sheeran (2004) found significant effects of certainty on attitude-behavior relationships across four studies with small-medium average effect sizes. Conner et al. (2022b) showed that a measure of certainty taken in relation to general thoughts and feelings about the behavior (e.g., How certain are you about what you think about wearing a face covering in public places? Not at all certain-Extremely certain’) in a multi-behavior study significantly moderated the attitude-behavior relationship (i.e., higher certainty associated with attitudes that were better predictors of behavior).

A limited number of studies have reported that intentions held with greater certainty better predict behavior (Bagozzi and Yi, 1989; Bassili, 1993; Pieters and Verplanken, 1995; Chandrashekaran et al., 2000; Sheeran, 2002; Sheeran and Abraham, 2003). A meta-analysis by Cooke and Sheeran (2004) reported that that certainty was a significant moderator of the intention-behavior relationship across two studies, with those with more certain intentions (*r* = 0.64) compared to those with less certain intentions (*r* = 0.41) showing stronger intention-behavior relationships. Sheeran and Abraham (2003) reported that intention certainty significantly moderated the intention-physical activity relationship.

Attitude *importance* is the degree to which an individual attaches significance to the attitude. This is a predictor of attitude strength that has received considerable attention (e.g., it is the focus of the first *Annual Review of Psychology* article focusing on attitude strength; Howe and Krosnick, 2017). Howe and Krosnick (2017) argue that attitude importance is a key predictor of attitude strength and reflects the degree of priority a person attaches to an attitude and distinguish it from concepts that link an attitude to one’s values or self-image (e.g., centrality, involvement, ego-involvement, salience, personal relevance). The most frequently used measures of this construct tap how important the attitude or object is to the individual, how concerned they are about it, or how deeply they care about it (i.e., meta-judgmental measures; Krosnick, 1989; Gopinath and Nyer, 2009). Studies show greater attitude importance to be associated with stronger attitude-behavior relationships in relation to product choices (Kokkinaki and Lunt, 1997), work behavior (Ziegler and Schlett, 2016), and environmental behaviors (Bolsen, 2013). There are fewer tests of the impact of attitude importance on attitude stability with mixed findings (Krosnick, 1988).

Eaton and Visser (2008) note that although typical definitions of attitude importance focus on the significance that people attach to their attitude toward a given object, measures of attitude importance (Boninger et al., 1995) tend to focus on how important the attitude object is to them. However, studies show that measures of these two aspects of attitude importance are extremely highly correlated (Boninger et al., 1995). Conner et al. (2022b) showed a measure of importance taken in relation to the attitude object or behavior (e.g., “How important is wearing a face covering in public places to you? Not at all – Extremely important”) in a multi-behavior study significantly moderated the attitude-behavior relationship (i.e., higher importance associated with better predictions of behavior).

Given the attention in the attitude domain it is perhaps surprising that importance has not received any attention in relation to intention strength. It might be expected that intentions toward behaviors judged to be important (or indeed behaviors judged to be important) might be stronger (i.e., durable and impactful) than those toward behaviors not judged to be important. Tests in the intention domain are warranted given the large amount of attention devoted to this variable in relation to attitude strength and the conclusion that it is a key predictor that may account for the role of other predictors (Conner et al., 2022b).

In the attitude domain, *vested interest* is the degree to which the attitude object is perceived to be of significant personal consequence (Crano, 1995; Howe and Krosnick, 2017). Crano (1995) notes the strong overlap between vested interest and personal relevance (and also attitude importance). Personal relevance is measured by simple, meta-judgmental measures about the attitude object anchored with “not personally relevant” to “personally relevant” (Haugtvedt and Wegener, 1994). To date there are no tests of vested interest or personal relevance taken in relation to the behavior as a moderator of the intention-behavior relationship. It might be expected that greater vested interest/personal relevance of a behavior would be associated with intentions that are more predictive of engaging in the behavior.

## Future directions

In this section two related directions for future research on the intention-behavior gap are set out based on the above review of the existing literature (see Figure 1 for a summary). These are the systematic study of individual and multiple moderators of the intention-behavior relationship and exploration of mechanisms by which moderators influence the intention-behavior relationship.

### Systematic study of individual and multiple moderators

The literature reviewed above has highlighted a wide range of moderators of the intention-behavior relationship which together could provide a better understanding of the intention-behavior gap in relation to physical activity and other behaviors. The moderators reviewed prominently included goal dimensions, intention strength predictors and intention stability but also those linked to the basis of intention, structure of intention, and the personality dimension of conscientiousness. The concept of intention strength might provide a useful way to conceptualize these various different moderators. In this view, the different individual moderators would be considered as predictors of intention strength. As such, it would be useful to explore their effects on the intention-behavior relationship as well as on other features of intention strength such as intention stability, pliability and impacts on information processing (for an example see Cooke and Sheeran, 2013). For example, in addition to the predictors of strength reviewed above (i.e., extremity, knowledge, accessibility, certainty, and importance), goal dimensions such as goal desire, goal commitment, goal priority and goal conflict and measures of the structure of intentions (e.g., motivational coherence) might all be expected to influence the intention-behavior relationship as well as other features of intention strength (i.e., intention stability, intention pliability and impact on information processing). As noted below, studies assessing the impact of moderators on both the intention-behavior relationship and other features of intention strength opens up the possibility of testing these other features as mechanisms to explain their effects on the intention-behavior relationship. In particular, research on intention stability as a mechanism to explain the effects of various intention-behavior moderators is reviewed below.

Studies examining multiple moderators of the intention-behavior relationship open up additional avenues for analysis. Relatively few studies have assessed more than one of these moderators, making comparisons of effects difficult due to differences in samples and behaviors. More studies could usefully assess multiple moderators to allow more direct comparisons of effects without the potential confounding factors that limit between study comparisons (e.g., sample, behavior or measure differences). Such studies could also allow exploration of the inter-relationships between moderators. In the attitude domain, a number of studies have examined the inter-relationships of different predictors of attitude strength. The general conclusion is that these predictors of attitude strength are both conceptually and empirically distinct (Luttrell and Sawicki, 2020). Correlations (e.g., Conner et al., 2022b) and confirmatory factor analyses (Krosnick et al., 1993; Lavine et al., 1998) support the idea that each constitutes its own latent factor), although they are intercorrelated. This may also be the case for moderators of the intention-behavior relationship although this remains to be determined. Studies that assess multiple intention-behavior moderators could employ exploratory and confirmatory factor analyses to test for underlying dimensions among moderators.

A limited number of studies have examined the effects of more than one predictor of attitude strength at a time on more than one feature of attitude strength (Bassili, 1996; Prislin, 1996; Luttrell and Togans, 2021; Conner et al., 2022b; see also Philipp-Muller et al., 2020 on predicting intentions). Such studies also allow exploration of the simultaneous effects of different predictors of attitude strength in order to assess if, for example, particular predictors dominate in their impact on the stability of attitudes and the attitude-behavior relationship. Conner et al. (2022b) showed that attitude certainty, importance, subjective knowledge, moral basis of attitude, cognitive-affective felt and potential ambivalence plus cognitive-affective inconsistency, but not attitude elaboration, individually and in combination (excluding potential ambivalence) predicted attitude stability. It was also found that attitude certainty, importance, subjective knowledge, moral basis of attitude, cognitive-affective felt ambivalence, cognitive-affective inconsistency plus attitude stability, but not cognitive-affective potential ambivalence or attitude elaboration, each individually moderated the attitude-behavior relationship. But when considered simultaneously only attitude importance and cognitive-affective inconsistency moderated the attitude-behavior relationship and only the former remained significant when controlling for attitude stability. This supports the idea that attitude importance is a key predictor of attitude strength (see Howe and Krosnick, 2017). Similar studies in relation to the various moderators of the intention-behavior relationship would be valuable to identify key moderators. This might indicate that moderators found to be dominant in relation to attitude strength such as importance are also key in relation to intention strength or whether different patterns exist (e.g., a different moderator such as certainty or several moderators are important).

### Exploration of mechanisms

A further useful direction for research on the intention-behavior gap would be exploration of the mechanisms by which moderators of this relationship have their effect. In statistical terms this would be a test of whether measures of a proposed mechanism fully or partially mediate the effect of a moderator of the intention-behavior relationship. As noted above, intention stability, intention pliability and impacts on information processing might each be features of intention strength that could explain the effects of various moderators of the intention-behavior relationship. For example, a moderator like conscientiousness might be associated with stronger intention-behavior relationships because conscientious individuals hold intentions that are more stable and less pliable in the face of persuasive attempts and also because they are more likely to denigrate information that conflicts with their existing intentions (or bolster information that is consistent with their existing intentions).

The stability of attitudes has particularly received attention as a mechanism to explain the effects of other moderators of the attitude-behavior relationship. The idea that attitude temporal stability is one important mechanism through which strong attitudes better predict behavior is known as the prediction explanation (Fabrigar et al., 2005). As Schwartz (1978) noted, attitudes are unlikely to predict subsequent behavior unless they remain stable over the intervening time interval between when the two are measured. For example, Conner et al. (2022b) showed that the moderating effects of certainty, importance, knowledge, moral basis of attitude, ambivalence and inconsistency on the attitude-behavior relationship were fully or partially explained by their effects on attitude stability. Other mechanisms relate to other features of attitude strength (e.g., changing/biased perceptions of the attitude object; Fabrigar et al., 2005).

Similarly, intention stability rather than being just another moderator of the intention-behavior relationship, may represent an important mechanism through which other moderators of the intention-behavior relationship have their effect. That is, a moderator like high goal commitment may strengthen the intention-behavior relationship *because* it is associated with more stable intentions. The extent to which intention stability effects fully mediate the effects of other moderators of the intention-behavior relationship would point to stability being a key mechanism by which they have this effect. Research has shown intention stability to fully or partially explain the effect of various other moderators of the intention-behavior relationship. For example, Sheeran and Abraham (2003) reported that intention stability moderated the intention–behavior relationship for exercising (intention-behavior correlation for low stability, *r*_+_ = 0.49, for high stability, *r*_+_ = 0.76). More importantly, Sheeran and Abraham (2003) found that intention stability fully mediated the effect of other moderators (i.e., intention certainty, past behavior, self-schema, anticipated regret and attitudinal control) of the intention–behavior relationship. This suggests that the mechanism by which these other moderators have their effect on intention–behavior relationships is through changing the temporal stability of intentions. Hence, factors that might be expected to make individual intentions more stable over time would be expected to increase the impact that these intentions have on behavior and so reduce the intention–behavior gap.

Further studies that consider intention stability and other mechanisms as mediators of the effects of moderators of the intention-behavior relationship would be valuable. This is the case both for the examination of the effects of individual (e.g., motivational coherence, Sheeran and Conner, 2017) and multiple (e.g., Sheeran and Abraham, 2003) moderators. In relation to other mechanisms, it was noted earlier that in the attitude strength domain, attitude stability was assumed not to be the only mechanism that might explain the effects of moderators of the attitude-behavior relationship. Similarly, in the intention domain, intention stability may not be the only mechanism to explain the effects of moderators of the intention-behavior relationship. For example, such moderators may have their effects on the intention-behavior relationship through their effects on various aspects of goal pursuit. Sheeran and Webb (2016) provide a useful review of the processes leading to goal realization. Self-regulatory challenges such as getting started, keeping goal pursuit on track, and bringing goal pursuit to a successful close are highlighted. Aspects of these processes could also form mechanisms explaining how moderators of the intention-behavior relationship have their effect (see also Johnson et al., 2006 for relevant suggestions from control theory). Relatedly, increased effort during goal pursuit and greater persistence in the face of obstacles could constitute additional mechanisms by which moderators have their effect on the intention-behavior relationship (see Bogg and Roberts, 2004, 2013 on conscientiousness). Studies that compare various different mechanisms through which individual and multiple moderators of the intention-behavior relationship have their effects could make an important contribution to understanding in this area. Further, experimental studies that attempt to manipulate moderators or mechanisms could aid our understanding of causal relationships in relation to intentions and behavior.

## Conclusion

This manuscript has reviewed the work on various moderators of the intention-behavior relationship in order to provide insights into the factors that might explain the gap between the two. The focus was on the concept of intention strength and how this might add to understanding in this area. In particular, the idea that strong intentions may not only better predict behavior but also be more stable over time was advanced and it was noted that stability may be an important mechanism by which moderators of the intention-behavior relationship have their effects. In addition, a number of potential moderators drawn from the concept of intention strength and its parallels to predictors of attitude strength were reviewed. Future research in this area could benefit from a systematic examination of multiple moderators of the intention-behavior relationship and the extent to which intention stability or other mechanisms might explain their moderating effects. Relatedly, future research should also consider other key features of intention strength such as the pliability of intentions and their impact on the processing of intention-relevant information. Although strong intentions may be stable over time and more predictive of engaging in behaviors such as physical activity, they may also be more difficult to change through intervention and may lead to the biased processing of messages designed to change them (see Johnson et al., 2006; Cooke and Sheeran, 2013).

## Author contributions

MC and PN developed the idea, contributed to and wrote the manuscript, and approved the submitted version.
